# Anesthesia for patients undergoing transsternal thymectomy for juvenile myasthenia gravis

**DOI:** 10.4103/1658-354X.76490

**Published:** 2011

**Authors:** Lianne Stephenson, Igor Tkachenko, Robert Shamberger, Christian Seefelder

**Affiliations:** *Department of Anesthesiology, University of Wisconsin, Madison WI, USA*; 1 *Department of Anesthesia & Critical Care, The University of Chicago, Chicago IL, USA*; 2 *Department of Surgery, Children’s Hospital Boston, Harvard Medical School, Boston MA, USA*; 3 *Department of Anesthesiology, Perioperative and Pain Medicine, Children’s Hospital Boston, Harvard Medical School, Boston MA, USA*

**Keywords:** *Epidural anesthesia*, *juvenile myasthenia gravis*, *pediatric anesthesia*, *remifentanil*

## Abstract

**Background:**

Juvenile myasthenia gravis (JMG) is the rare form of myasthenia gravis presenting in childhood and adolescence. When medical management fails, thymectomy is offered for these patients. Complete resection of the thymus is best achieved through transsternal thymectomy. Anesthetic management of patients with JMG is challenging, particularly in regards to the goals of postoperative pain control, respiratory function, and extubation.

**Methods:**

We retrospectively reviewed the medical records of 13 patients, ranging in age from 6 to 22 years, who underwent transsternal thymectomy for JMG. Information on patient demographics, characteristics of their disease and treatment, anesthetic management, and postoperative course were collected.

**Results:**

All patients had undergone multiple treatment modalities and presented for surgery because of inadequate symptom control with medical management. As expected for a pediatric population, anesthesia induction was age dependent. 40% of the patients underwent an inhalation induction and 60% underwent an intravenous induction. Anesthesia was maintained with a low-dose inhalation agent in all patients, supplemented in 84% of patients with a remifentanil infusion, and in 69% of patients with an epidural infusion. Muscle relaxants were avoided in all patients. With this regimen, 92% of patients could be extubated successfully in the operating room.

**Conclusion:**

We found that avoidance of muscle relaxants and use of remifentanil with a low-dose hypnotic agent provided a stable intraoperative course, facilitated rapid emergence, and allowed early extubation in patients with JMG undergoing transsternal thymectomy. Epidural analgesia reduced the need for intra- and postoperative intravenous opioids and did not have an adverse effect on respiratory strength.

## INTRODUCTION

Juvenile myasthenia gravis (JMG) is a rare autoimmune disorder resulting in muscle weakness. It is an antibody-mediated disease affecting the postsynaptic neuromuscular junction, resulting in the blockade of synaptic transmission. Immune-mediated injury results in a myriad of clinical manifestations that can include ocular, bulbar, and generalized muscle weakness. Of all patients with myasthenia gravis (MG), 10 to 20% are infants and children who can present with congenital, transient neonatal, or most commonly JMG. Most large series describing the anesthetic management of MG are on adult patients.[1] Although the adult and juvenile forms of MG share a similar pathophysiology and clinical presentation, the epidemiology, prognosis, and some aspects of treatment are different for JMG.[2] Apart from an older series,[3] most reports on the current anesthetic management in children are case reports.[4] We present a case series of the perioperative management of patients with JMG undergoing transsternal thymectomy.

## METHODS

Medical records of patients undergoing transsternal thymectomy for management of JMG at our institution between 2000 and 2009 were reviewed retrospectively. Patients without MG undergoing thymectomy for other indications were not included. This study was approved by the hospital’s Committee for Clinical Investigation and consent requirement was waived. Interest was only in the perioperative course, not in the long-term medical outcome of the surgery. Information obtained included patient demographics (age, weight, gender, symptoms, antibody status, and previous medical management), anesthetic management aspects (use of induction and maintenance agents, muscle relaxants, opioids and anticholinesterases, epidural analgesia), and perioperative course (extubation success, postoperative analgesia, postoperative plasmapheresis, intensive care unit duration, and overall length of hospital stay).

## RESULTS

A total of 13 patients were identified, ranging from 6 to 22 years (mean, 13.2 years) in age and from 21 to 79 kg (mean, 50.3 kg) in weight. Patient demographics, clinical symptoms, and preoperative medical management are summarized in detail in Table 1. JMG was diagnosed 3 months to 6 years before surgery. Ocular symptoms were the most common presentation. Because generalized muscle weakness was uncommon, only a few patients had perioperative pulmonary function tests performed. All patients had undergone multiple treatment modalities and presented for surgery because of inadequate symptom control with medical treatment. Twelve patients were antibody-positive. One patient (patient 5) had JMG and a thymoma. One patient (patient 12) developed MG symptoms after a previous thymectomy for thymoma and underwent repeat sternotomy for resection of residual thymic tissue. Patients were continued on their routine schedule of pyridostigmine until the morning of surgery. Patients on long-term steroids as part of their MG management received supplemental steroids perioperatively to account for potential adrenal suppression. Ten patients underwent preoperative plasmapheresis as part of the preoperative regimen to optimize their status.

**Table 1:** Patient characteristics

Patient	Age (years)	Weight (kg)	Gender	Duration of symptoms	Symptoms o/b/g	Antibodies	Medical management	Preoperative plasmapheresis
1	10	40	F	6 months	o/g	Positive	Pyridostigmine(180mg tid, 60mg tid), Steroid, IVIg	Yes
2	8	32	F	6 years	o	Positive	Steroid only	No
3	15	63	F	3 months	b/g	Positive	Pyridostigmine (90mg every 4 h), IVIg	Yes
4	18	75	F	3 months	o/b/g	Positive	Pyridostigmine (60mg three times daily)	Yes
5	15	68	M	5 months	o/b/g	Positive	Pyridostigmine (60mg every 6 h)	Yes
6	16	52	F	1 year	o/b/g	Positive	Pyridostigmine (30mg every 6 h), IVIg	No
7	17	79	F	4 months	o/b/g	Positive	Pyridostigmine (45mg every 6 h)	Yes
8	18	59	F	1 year	o/b/g	Positive	Pyridostigmine (60mg every 6 h)	Yes
9	9	43	M	6 months	o/b/g	Positive	Pyridostigmine (60mg every 4 h), Steroid, IVIg,	Yes
10	6	21	M	2 years	o/g	Negative	Pyridostigmine (15mg every 12 h/30mg every 12 h), IVIg	No
11	12	43	F	5 months	o/b/g	Positive	Pyridostigmine (60mg every 6 h)	Yes
12	6	26	M	1 month	o/g	Positive	Pyridostigmine (40mg every 4 h), IVIg	Yes
13	22	53	F	12 months	o/b	Positive	Pyridostigmine (60mg every 6 h)	Yes

Gender: f = Female, m = Male. Symptoms: o = Ocular, b = Bulbar, g = Generalized weakness, IVIg = Intravenous immunoglobulin

Table 2 lists the anesthetic and postoperative details. Anesthesia was conducted at the discretion of the attending anesthesiologist. Standard ASA monitoring was used in all patients and eleven patients had invasive arterial blood pressure monitoring. Five children underwent inhalation induction and the remaining eight patients had an intravenous induction. None of the patients received muscle relaxants for intubation or during surgery. Before intubation, 4% topical lidocaine was applied to the vocal cords and trachea in most patients. A remifentanil infusion was started before intubation in eleven patients, one of whom developed self-limited bradycardia during intubation. All patients were offered epidural analgesia. Nine patients consented and underwent successful epidural catheter placement. This was done either awake-sedated prior to or asleep after induction of anesthesia, depending on the patient’s age and ability to cooperate with the epidural catheter placement. Anesthesia was maintained with low-dose inhalation agent and remifentanil or other IV opioids. If an epidural catheter had been placed, it was used as part of the anesthetic. Pyridostigmine was given to six patients intraoperatively. Blood loss was minimal and no patient required a blood transfusion. Upon completion of surgery, patients were awakened and breathing efforts and muscle strength were assessed clinically by tidal volume, strength of cough, hand grip, and head lift. Patients with preoperative generalized muscle weakness reported decreased strength in the immediate postoperative period and clinically demonstrated muscle weakness. Twelve patients were extubated in the operating room. One teenager (patient 3) indicated that upon awakening, she felt uncomfortable breathing without support; she was extubated 9 hours later in the intensive care unit (ICU). All patients were electively admitted to the ICU after surgery. One patient (patient 12) with severe preoperative weakness required postoperative bi-level positive airway pressure (BIPAP) support. Initial postoperative pain control was achieved with epidural analgesia in seven patients, and two patients had combined epidural local anesthetic and intravenous morphine patient-controlled analgesia (PCA). In four patients, postoperative pain control was achieved with intravenous opioid PCA only.

**Table 2:** Intra and postoperative course summary

Patient	Preop sedation	Induction	Muscle relaxant	Maintenance	Remifentanil mcg/kg/min	Opioid intraop	Epidural level	AChEl intraop	Ex-tubation	Postop analgesia	Epi-dural days	Postop plasmapheresis	ICU days	Hospital day
1	None	8% Sevoflurane	No	0.4-0.8% Isoflurane 70% nitrous	0.5	None	T5/6	Neostigmine 2img	OR	Epidural (BUP/HM)	2	Yes	2	5
2	None	8% Sevoflurane Propofol 6omg	No	0.8-1.2% Isoflurane	0.4	Morphine	none	No	OR	Morphine PCA	-	No	2	3
3	Midazolam lmg (IV)	Propofol 200mg Fentanyl 250mcg	No	0.4-1% Isoflurane 70% nitrous	0.25	Fentanyl, morphine	none	Pyridostigmine 3mg	9 hrs postop	Morphine PCA	-	Yes	1	4
4	Midazolam 2mg (IV)	Thiopental 125mg Propofol isomg	No	0.4-0.6% Isoflurane 70% nitrous	0.1-0.3	Fentanyl, morphine	none	Neostigmine 3-5mg	OR	Morphine PCA	-	No	1	4
5	Midazolam 4mg (IV)	Thiopental 125img Fentanyl l00mcg	No	1-1.5% Isoflurane	None	Fentanyl	T4/5	Pyridostigmine 2mg	OR	Epidural (BUP) Morphine PCA	5	Yes	1	8
6	None	8% Sevoflurane Thiopental 125mg Propofol 200mg	No	1.9-2.2% Sevoflurane propofol (150-200mcg/kg/min)	0.1-0.3	None	T5/6	Neostigmine 0.25mg (given twice)	OR	Epidural (BUP/HM)	3	No	1	4
7	Midazolam 4mg (IV)	Propofol 250mg Fentanyl l00mcg	No	<0.7% Sevoflurane 50% nitrous	0.1-0.2	Fentanyl, morphine	T6/7	No	OR	Epidural (BUP/ HM/C)	2	Yes	1	4
8	Midazolam 2mg (IV)	Propofol 150mg Fentanyl 25mcg	No	<1% Sevoflurane 50% nitrous	0.2	Fentanyl	T 9/10	No	OR	Epidural (BUP/HM)	3	Yes	2	6
9	Midazolam 2omg (po)	8% Sevoflurane	No	1-1.8% Sevoflurane 70% nitrous	0.15	None	T7/8	No	OR	Epidural (BF) Morphine PCA	2	Yes	1	7
10	None	8% Sevoflurane	No	<1.5% Isoflurane 50% nitrous	0.2	None	T8/9	Pyridostigmine lmg	OR	Epidural (BUP/HM)	3	No	1	4
11	None	Thiopental 50mg Propofol 200mg Fentanyl l00mcg	No	0.8% Isoflurane propofol (l00mcg/ kg/min)	0.2	Fentanyl	T5/6	No	OR	Epidural (BF changed to BFC)	3	Yes	1	5
12	Midazolam 2mg (IV)	Propofol l00mg	No	1.2% Isoflurane 50% nitrous	None	Fentanyl, morphine	T7/8	No	OR	Epidural (BFC)	5	Yes	4	9
13	Midazolam 6mg (IV)	Propofol 200mg Fentanyl l00mcg	No	<2% Sevoflurane	0.2	Fentanyl, morphine	None	No	OR	Morphine PCA	-	Yes	3	5

AChEI=acetylcholinesterase inhibitor. PCA=patient controlled opioid analgesia. BUP=bupivacaine 0.1%, HM=hydromorphone 10mcg/ml, C=clonidine 0.4mcg/ml, F=fentanyl 2mcg/ml

## DISCUSSION

JMG can affect children as young as 1 year of age.[2] There is a female predominance in patients of 10 years and older, which is similar to the adult form, but gender discrepancy does not exist for the younger children with JMG.[2] Fluctuating motor weakness is the hallmark of the disease. Prepubertal JMG is more likely to have isolated ocular symptoms such as ptosis and diplopia, whereas generalized muscle weakness and fatigability is more common in the postpubertal presentation. However, children with JMG tend to have fewer generalized symptoms compared with adults.[2] Patients may also have proximal limb weakness or bulbar symptoms, which affects their ability to chew, swallow, and articulate. More serious symptoms involve weakness of the diaphragm, which may result in respiratory distress.

Although JMG is typically a clinical diagnosis, antibody studies can help confirm the diagnosis. 80% of adult patients will test positive for acetylcholine receptor antibodies as opposed to 50% of prepubertal cases of JMG.[2] Seronegative patients may test positive to autoantibodies targeting muscle-specific kinase. These patients also have disruption at the postsynaptic neuromuscular junction, but it is caused by a different antigen. Further testing includes the edrophonium test, repetitive nerve stimulation, and single fiber electromyography. Chest radiograph and computed tomography are performed to investigate for the presence of a thymoma which occurs less commonly in JMG than in adult MG. Cooperative patients with generalized weakness should undergo pulmonary function testing preoperatively and postoperatively.

Medical management depends on the severity of JMG and the response to treatment. The clinical course of JMG can be fluctuating with intermittent remissions and exacerbations. Children tend to have higher rates of spontaneous remission than adults.[2] Pyridostigmine, a long-acting acetylcholine esterase inhibitor (AChEI), is the first-line therapy. It prevents acetylcholine breakdown, and subsequently increases the synaptic acetylcholine concentration which improves neuronal transmission. Patients with progressive symptoms are treated with high-dose steroids and other immunosuppressant drugs such as cyclosporine and azathioprine.[5] Steroids are often used cautiously in children because of the long-term side effects. Intravenous immunoglobulin (IVIg) and plasmapheresis frequently provide symptomatic improvement which is utilized perioperatively and during exacerbations and myasthenic crisis. Laboratory perturbations seen with plasmapheresis that may affect the perioperative course include coagulopathies, anemia, hypoalbuminemia, and hypocalcemia. Unfortunately, IVIg and plasmapheresis seem to have poor long-term benefits for most patients.[5]

Patients resistant to medical treatment can be offered thymectomy, which may decrease morbidity from JMG.[6] Hyperplastic thymus, not thymoma, is the most common abnormality found in children with JMG who have undergone thymectomy. Unlike adults, thymectomy within 2 years of presentation may result in a higher rate of remission in Caucasian children.[2] Thymectomy can be performed through a transcervical, thoracoscopic, or transsternal approach. Complete resection of the thymus is best achieved through transsternal thymectomy, as thymic tissue may be widely distributed in the pretracheal and anterior mediastinal fat.[7]

Based on the literature, no particular anesthetic technique has proven superior for thymectomy in JMG. A variety of inhalational and intravenous techniques have been used successfully. Total intravenous anesthesia avoids the muscle relaxing effect of inhalational agents[8] and may facilitate early extubation,[9] but does not have clinical advantages.[10,11] Muscle relaxants are not generally contraindicated but should be used with caution. Due to the decreased receptor density, patients with JMG are resistant to succinylcholine. Although an increased succinylcholine dose may be required, its duration of action may be prolonged in the presence of AChEIs. Patients with JMG are sensitive to nondepolarizing muscle relaxants, which must be administered in reduced doses and titrated carefully. We prefer to avoid the use of muscle relaxants. Remifentanil is a useful adjunct to help achieve an adequate anesthetic depth for tracheal intubation and to prevent intraoperative patient movement, while facilitating the rapid return of adequate respiratory function for emergence.[4,12] We did not notice a prolonged duration of action for remifentanil, which is a concern in patients with JMG taking AChEIs or with decreased levels of plasma esterases from preoperative plasmapheresis.[13]

Successful use of regional anesthesia has been reported for patients with MG. Epidural analgesia has been used for transsternal thymectomy both with[4,12] and without[14] general anesthesia. It reduces the need for intraoperative and postoperative systemic opioids and may promote early extubation.[15] We did not see a detrimental effect of epidural local anesthetic use on respiratory muscle strength. Ester local anesthetics should be avoided in patients taking AChEIs.

There are no clear guidelines regarding perioperative administration of AChEIs. Older reports indicated increased postoperative complications with perioperative AChEI administration and recommended avoiding them preoperatively.[3] However, advising patients to withhold their preoperative dose may result in increased muscle weakness. In contrast, patients who continue to take their pyridostigmine may have increased secretions or develop a cholinergic crisis with muscarinic symptoms (nausea, sweating, miosis, and bradycardia). Severe bradycardia is a concern in patients on AChEIs, with anesthetic techniques utilizing remifentanil,[16] high thoracic epidural local anesthetics,[17] or spinal anesthesia,[18] particularly with vagal stimulation occurring during intubation. As reported by others,[3] one of our patients developed severe but self-limited sinus bradycardia during intubation. For subsequent cases, we administered an anticholinergic agent preemptively. We give the patient’s routine AChEI dose up to the time of surgery. We no longer administer an intravenous AChEI dose during surgery, but wait until a postoperative clinical evaluation is possible and the patient can resume oral medication.

The need for high preoperative doses of pyridostigmine and reduced preoperative pulmonary function was associated with postoperative respiratory complications in patients with MG.[19–21] Similarly, all of our patients with no or only mild systemic weakness were extubated successfully in the operating room and had no perioperative problems related to their disease or the anesthetic. Patients with more severe systemic weakness reported increased weakness with emergence, despite the avoidance of muscle relaxants. Only one patient required brief postoperative ventilation. All of our patients were taken to the ICU for postoperative monitoring, pain control, plasmapheresis, and respiratory support with oxygen and BIPAP, if needed.

In conclusion, we found that the avoidance of muscle relaxants and use of remifentanil and a low-dose hypnotic agent can provide a stable intraoperative course, rapid emergence, and early extubation in patients with JMG undergoing transsternal thymectomy. Adequate postoperative analgesia can be achieved with the use of epidural analgesia, without detrimental effect on postoperative recovery.

## References

[1] El-Dawlatly A, Turkistani A, Alkattan K, Hajjar W, Delvi MB, Alshaer A (2008). Anesthesia for thymectomy in myasthenia gravis--a report of 115 cases. Middle East J Anesthesiol.

[2] Chiang LM, Darras BT, Kang PB (2009). Juvenile myasthenia gravis. Muscle Nerve.

[3] Davies DW, Steward DJ (1973). Myasthenia gravis in children and anaesthetic management for thymectomy. Can Anaesth Soc J.

[4] Bagshaw O (2007). A combination of total intravenous anesthesia and thoracic epidural for thymectomy in juvenile myasthenia gravis. Paediatr Anaesth.

[5] Gadient P, Bolton J, Puri V (2009). Juvenile Myasthenia Gravis: Three Case Reports and a Literature Review. J Child Neurol.

[6] Tracy MM, McRae W, Millichap JG (2009). Graded response to thymectomy in children with myasthenia gravis. J Child Neurol.

[7] Gronseth GS, Barohn RJ (2000). Practice parameter: Thymectomy for autoimmune myasthenia gravis (an evidence-based review): Report of the Quality Standards Subcommittee of the American Academy of Neurology. Neurology.

[8] Nitahara K, Sugi Y, Higa K, Shono S, Hamada T (2007). Neuromuscular effects of sevoflurane in myasthenia gravis patients. Br J Anaesth.

[9] Ng JM (2006). Total intravenous anesthesia with propofol and remifentanil for video-assisted thoracoscopic thymectomy in patients with myasthenia gravis. Anesth Analg.

[10] Della Rocca G, Coccia C, Diana L, Pompei L, Costa MG, Tomaselli E (2003). Propofol or sevoflurane anesthesia without muscle relaxants allow the early extubation of myasthenic patients. Can J Anaesth.

[11] Gritti P, Carrara B, Khotcholava M, Bortolotti G, Giardini D, Lanterna LA (2009). The use of desflurane or propofol in combination with remifentanil in myasthenic patients undergoing a video-assisted thoracoscopic-extended thymectomy. Acta Anaesthesiol Scand.

[12] Mekis D, Kamenik M (2005). Remifentanil and high thoracic epidural anaesthesia: a successful combination for patients with myasthenia gravis undergoing transsternal thymectomy. Eur J Anaesthesiol.

[13] Baraka AS, Haroun-Bizri ST, Gerges FJ (2004). Delayed postoperative arousal following remifentanil-based anesthesia in a myasthenic patient undergoing thymectomy. Anesthesiology.

[14] Tsunezuka Y, Oda M, Matsumoto I, Tamura M, Watanabe G (2004). Extended thymectomy in patients with myasthenia gravis with high thoracic epidural anesthesia alone. World J Surg.

[15] Chevalley C, Spiliopoulos A, de Perrot M, Tschopp JM, Licker M (2001). Perioperative medical management and outcome following thymectomy for myasthenia gravis. Can J Anaesth.

[16] Nauphal M, Baraka A (2008). Bradycardia, hypotension and bronchospasm following remifentanil-propofol in a myasthenic patient treated by pyridostigmine. Middle East J Anesthesiol.

[17] Lin TC, Hsu CH, Kong SS, Cherng CH, Wong CS (2002). Ventricular asystole and complete heart block after thoracic epidural analgesia for thymectomy. Eur J Anaesthesiol.

[18] Inoue S, Shiomi T, Furuya H (2002). Severe bradycardia in a patient with myasthenia gravis during transurethral ureterolithotripsic procedure under spinal anaesthesia. Anaesth Intensive Care.

[19] Naguib M, El Dawlatly AA, Ashour M, Bamgboye EA (1996). Multivariate determinants of the need for postoperative ventilation in myasthenia gravis. Can J Anaesth.

[20] Leventhal SR, Orkin FK, Hirsh RA (1980). Prediction of the need for postoperative mechanical ventilation in myasthenia gravis. Anesthesiology.

[21] Mori T, Yoshioka M, Watanabe K, Iwatani K, Kobayashi H, Terasaki H (2003). Changes in respiratory condition after thymectomy for patients with myasthenia gravis. Ann Thorac Cardiovasc Surg.

